# Balanced nutrient requirements for maize in the Northern Nigerian Savanna: Parameterization and validation of QUEFTS model

**DOI:** 10.1016/j.fcr.2019.107585

**Published:** 2019-09-01

**Authors:** Bello M. Shehu, Bassam A. Lawan, Jibrin M. Jibrin, Alpha Y. Kamara, Ibrahim B. Mohammed, Jairos Rurinda, Shamie Zingore, Peter Craufurd, Bernard Vanlauwe, Adam M. Adam, Roel Merckx

**Affiliations:** a Department of Earth and Environmental Sciences, Division of Soil and Water Management, KU Leuven, Kasteelpark Arenberg 20, 3001 Heverlee, Belgium; b Department of Soil Science, Bayero University Kano, Kano 70001, Nigeria; c Centre for Dryland Agriculture (CDA), Bayero University Kano, Kano 70001, Nigeria; d International Institute of Tropical Agriculture, PMB 5320, Oyo Road, Ibadan 200001, Oyo State, Nigeria; e International Plant Nutrition Institute (IPNI), c/o IFDC—East & Southern Africa Division, ICIPE Compound, Duduville—Kasarani, Thika Road, Nairobi, P.O. Box 30772-00100, Kenya; f International Maize and Wheat Improvement Center (CIMMYT), CIMMYT South Asia Regional Office, NARC Research Station, Khumaltar, Lalitpur, Kathmandu, Nepal

**Keywords:** Site-specific fertilizer recommendations, Indigenous nutrient supply, Soil fertility variability, QUEFTS model, *Zea mays* L.

## Abstract

Establishing balanced nutrient requirements for maize (*Zea mays* L.) in the Northern Nigerian Savanna is paramount to develop site-specific fertilizer recommendations to increase maize yield, profits of farmers and avoid negative environmental impacts of fertilizer use. The model QUEFTS (QUantitative Evaluation of Fertility of Tropical Soils) was used to estimate balanced nitrogen (N), phosphorus (P) and potassium (K) requirements for maize production in the Northern Nigerian Savanna. Data from on-farm nutrient omission trials conducted in 2015 and 2016 rainy seasons in two agro-ecological zones in the Northern Nigerian Savanna (i.e. Northern Guinea Savanna “NGS” and Sudan Savanna “SS”) were used to parameterize and validate the QUEFTS model. The relations between indigenous soil N, P, and K supply and soil properties were not well described with the QUEFTS default equations and consequently new and better fitting equations were derived. The parameters of maximum accumulation (*a*) and dilution (*d*) in kg grain per kg nutrient for the QUEFTS model obtained were respectively 35 and 79 for N, 200 and 527 for P and 25 and 117 for K in the NGS zone; 32 and 79 for N, 164 and 528 for P and 24 and 136 for K in the SS zone; and 35 and 79 for N, 199 and 528 for P and 24 and 124 for K when the data of the two zones were combined. There was a close agreement between observed and parameterized QUEFTS predicted yields in each of the agro-ecological zone (R^2^ = 0.69 for the NGS and 0.75 for the SS). Although with a slight reduction in the prediction power, a good fit between the observed and model predicted grain yield was also detected when the data for the two agro-ecological zones were combined (R^2^ = 0.67). Therefore, across the two agro-ecological zones, the model predicted a linear relationship between grain yield and above-ground nutrient uptake until yield reached about 50 to 60% of the yield potential. When the yield target reached 60% of the potential yield (i.e. 6.0 t ha^−1^), the model showed above-ground balanced nutrient uptake of 20.7, 3.4 and 27.1 kg N, P, and K, respectively, per one tonne of maize grain. These results suggest an average NPK ratio in the plant dry matter of about 6.1:1:7.9. We concluded that the QUEFTS model can be widely used for balanced nutrient requirement estimations and development of site-specific fertilizer recommendations for maize intensification in the Northern Nigerian Savanna.

## Highlights

• New and better fitting QUEFTS’ prediction equations for indigenous soil N, P, and K supply were developed for the Northern Nigerian Savanna.
• A good correlation was observed between the observed and parameterized QUFETS predicted maize grain yield.
• The QUEFTS model predicted a balanced N, P, K uptake to linearly increase with grain yield until 50-60% of the potential yield.
• The QUEFTS model is a suitable tool for site-specific nutrient recommendations in maize in the Northern Nigerian Savanna.

## 1 Introduction

The average number of individuals facing food insecurity in Nigeria has increased from 40.7 million between 2014 and 2016 to 46.1 million between 2015 and 2017 (FAOSTAT, 2018a). Maize (*Zea mays* L.), the most widely grown arable crop (Adesoji et al., 2016) and valuable cereal in Nigeria (FAO, 2016), can play a vital role in achieving food security in the country providing that the current meagre yield of the crop is increased drastically. Grain yield of maize in Nigeria over the last several decades has been hovering at 2 tonnes per hectare (t ha^−1^) (FAOSTAT, 2018b), which is far less than the yield of about 7 t ha^−1^ observed in well-managed field experiments (Fakorede and Akinyemiyu, 2003; Sileshi et al., 2010). One of the plausible reasons for the huge maize yield gap in Nigeria, as in other many countries in Sub-Saharan Africa, is poor soil fertility, the result of inherently low soil nutrient reserves as well as continuous cropping with inadequate nutrient replenishment (Manu et al., 1991; Ekeleme et al., 2014).

The Northern Nigerian Savanna (especially the Northern Guinea Savanna agroecology) is the most suitable zone for maize production in Nigeria due to high incident solar radiation, adequate rainfall, moderate incidences of biotic stresses and natural dryness at the time of harvest. However, soils in the Northern Nigerian Savanna are the major limitation for intensification of maize production. They are predominantly sandy Lixisols, Acrisols, and Cambisols with low activity clays (like kaolinite), small organic matter contents and small nutrient reserves, and prone to water and wind erosion (FDALR, 1999; FFD, 2012; Jones and Wild, 1975). Use of Fertilizer in maize production is necessary in this environment to replenish nutrients removed through the harvested product and exported crop residues (a common practice by most farmers in the area). Fertilizer use for maize production in the Northern Nigerian Savanna as the case in other agroecological zones of Nigeria, has been conventionally promoted through blanket recommendations regardless of wide variability in soil, climate and management regimes. The use of blanket fertilizer recommendations, however, is bound to create imbalanced crop nutrition since maize is cultivated in highly heterogeneous fields (Kihara et al., 2016; Shehu et al., 2018). Such imbalances lead to increased nutrient losses and low fertilizer use efficiency (Cassman et al., 2002), which can impede productivity, profitability and sustainability of a farm (Ezui et al., 2016). To reduce the persistent maize yield gaps in the Northern Nigerian Savanna, appropriate fertilizer recommendations need to be developed based on establishing balanced nutrient requirements, for specific yield targets and tailored to account for a specific field and/or soil condition.

A balanced requirement of a given nutrient refers to an amount of the nutrient required to meet a plant’s needs while maximizing the use efficiency of the nutrient (Ezui et al., 2016). When more than one nutrient is needed, for example, nitrogen (N), phosphorus (P) and potassium (K), balanced requirements refer to optimization of use efficiency of these three nutrients and simultaneously resulting in the largest response to their supplies (Ezui et al., 2016). The QUantitative Evaluation of the Fertility of Tropical Soils (QUEFTS) is a practical model that can be used to estimate balanced nutrient requirements for a location and for a target yield level while accounting for the interactions among macronutrients (particularly N, P and K) that affect plant’s physiological efficiencies (Janssen et al., 1990). The original QUEFTS model was developed for maize using data from Suriname and Kenya (Janssen et al., 1990) and it was later improved by Smaling and Janssen (1993) and Sattari et al. (2014). The QUEFTS model has been successfully tested for other crops like rice, wheat, cassava and sweet potato in different regions (Witt et al., 1999; Pathak et al., 2003; Ezui et al., 2016; Lam et al., 2016). Four major steps are involved in QUEFTS modelling (Sattari et al., 2014); (i) potential supply of the available nutrients (N, P and K) is calculated depending on the indigenous soil supply of the nutrient, plus average fertilizer recovery fraction multiplied by the amount of nutrient input. The indigenous soil nutrient supply is estimated by applying relations between soil chemical properties of the 0–20 cm soil layer and dry matter uptake of the nutrient in plots where this very nutrient is omitted; (ii) actual uptake of each nutrient is calculated based on the potential supply of that nutrient, considering the potential supply of the other two nutrients; (iii) the establishment of yield ranges as a function of uptake of the nutrients for maximum dilution and accumulation of that nutrient, respectively; and (iv) the yield ranges are combined into pairs, and yield estimated for pairs are averaged to obtain an ultimate yield estimate considering the maximum potential yield of the crop.

The most fickle part of QUEFTS model is the relations between soil chemical characteristics and the supply of available nutrients described in step 1 (i) above, as many local environmental factors may interfere (Sattari et al., 2014). In the original version of QUEFTS model the soil supply of available nutrients is calculated from soil chemical characteristics using regression equations primarily requiring datasets of soil organic carbon, available P, exchangeable K and pH (Janssen et al., 1990). The applicability and effectiveness of these default QUEFTS indigenous soil nutrient supply equations in different environments other than those which the model was developed is uncertain. Tabi et al. (2008) applied the QUEFTS model in maize to quantify potential supply of soil N and P, utilization efficiency and fertilizer recovery fractions in Northern Nigeria. This study was based on experiments conducted in only 27 farmers’ fields in two villages, limiting their representativeness for the entire maize producing area in the Northern Nigerian Savanna. It follows that it remains necessary to parameterize and validate the QUEFTS model to obtain balanced nutrient requirements for maize production at scale in the Northern Nigerian Savanna to enable effective implementation of site-specific nutrient management (SSNM) practices. The objectives of this study were to: (1) assess the relation between indigenous soil nutrient supply and soil chemical characteristics in the Northern Nigerian Savanna, (2) parametrize standard coefficients of QUEFTS model to determine balanced nutrient requirements for maize in the Northern Nigerian Savanna, and (3) validate the performance of the QUEFTS model in predicting maize grain yield in the Northern Nigerian Savanna.

## 2 Materials and methods

### 2.1 Site selection, description and experimental design

**Fig. 1:**
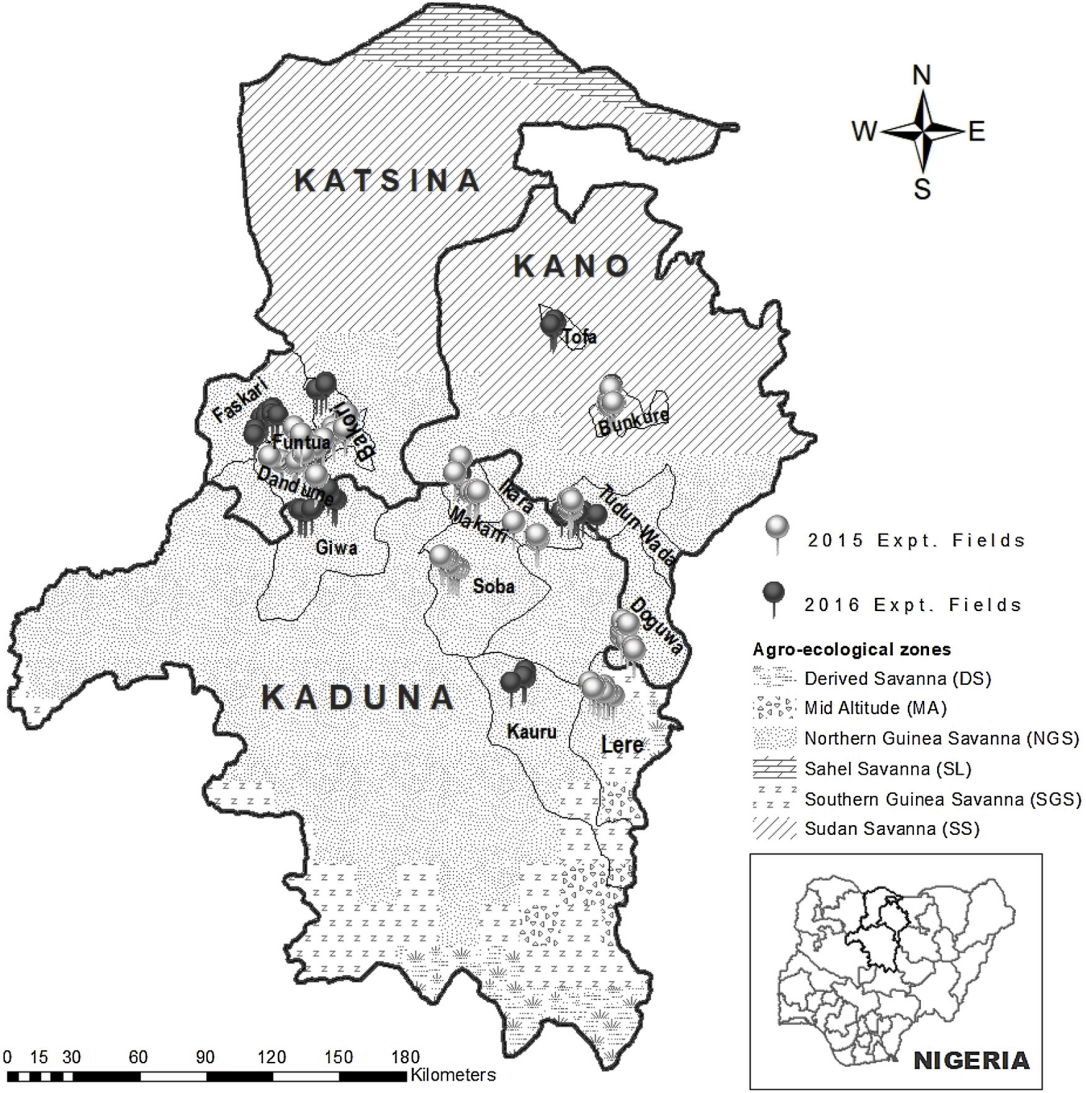
A map of Nigeria showing agroecological zones (AEZ), study sites and experimental fields for on-farm diagnostic nutrient omission trials (NOTs) established in 2015 and 2016 cropping seasons.

**Fig. 2:**
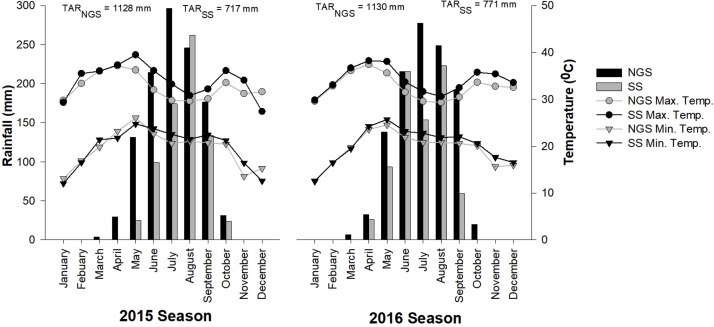
Annual rainfall, daily minimum and maximum temperatures of the two studied agroecological zones recorded in two cropping seasons (2015 and 2016). NGS: Northern Guinea Savanna; SS: Sudan Savanna; TAR_NGS_: total annual rainfall in NGS; TAR_SS_: total annual rainfall in SS; Min.: minimum; Max.: maximum; Temp.: temperature.

To generate datasets for this study, on-farm nutrient omission experiments were conducted over two rainy seasons (2015 and 2016) across fourteen study sites in three administrative States of the Northern Nigerian Savanna (Shehu et al., 2018). The three administrative States included Kaduna (with experimental fields in Lere, Kauru, Soba, Ikara, Makarfi, and Giwa local government areas), Katsina (with experimental fields in Funtua, Dandume, Faskari and Bakori local government areas) and Kano (with experimental fields in Tofa, Bunkure, Tudun Wada and Doguwa local government areas) (Fig. 1). The study sites were chosen to cover a broad range of maize growing conditions across the high production potential areas in the Northern Nigerian Savanna and to involve areas where research for development can support extension support programmes engaged in maize value chain initiatives. Overall the study sites fell within two agro-ecological zones i.e. the Northern Guinea Savanna (NGS) and Sudan Savanna (SS) (Fig. 1). The weather conditions of the two agro-ecological zones during the two years of experimentation are summarized in Fig. 2. The total annual rainfall in NGS was 1128 mm in 2015 and 1130 mm in 2016; total annual rainfall in SS was 717 mm in 2015 and 771 mm in 2016. Experimental fields were selected by generating one or two 10 km × 10 km grid(s) in each study site (depending on the size of the study site) using ArcGIS software (Environmental System Research Institute, Redlands, CA, USA). Within each of these 10 km × 10 km grid(s), five 1 km × 1 km sub-grids were delineated evenly. In each of the 1 km × 1 km sub-grids, a field for experimentation was randomly selected, considering the willingness of a farmer and availability of land for the trial setup. A total of ninety-five (95) and one hundred and three (103) experimental fields were selected in the 2015 and 2016 rainy seasons, respectively (Fig. 1). At each experimental field, two sets of trials were established side by side; one with hybrid maize (hybrid) and the other one with open-pollinated maize (OPV).

The nutrient omission experiments were composed of six nutrient application treatments: (i) control without nutrients applied (control), (ii) N omitted with P and K applied (-N), (iii) P omitted with N and K applied (-P), (iv) K omitted with N and P applied (-K), (v) treatment with all the three nutrients applied (NPK), and (vi) a treatment where secondary macronutrients (S, Ca and Mg) and micronutrients (Zn and B) were applied in addition to the NPK (NPK+). Primary macronutrients were applied at 140 kg N ha^−1^, 50 kg P ha^−1^ and 50 kg K ha^−1^ at each site in the NGS; and at 120 kg N ha^−1^, 40 kg P ha^−1^ and 40 kg K ha^−1^ at each site in the SS. The secondary macro- and micro-nutrients were applied at 24 kg S ha^−1^, 10 kg Ca ha^−1^, 10 kg Mg ha^−1^,5 kg Zn ha^−1^ and 5 kg B ha^−1^ at each site across the agro-ecological zones. Nitrogen (N) was applied in three equal splits, i.e. at planting (basal application), at 21 and 42 days after emergence (DAE), while all other nutrients were applied at planting. The open-pollinated maize varieties used were IWD C2 SYN F2 (with 105–110 days to maturity) and EVDT W STR (with 90–95 days to maturity) in the NGS and the SS study sites, respectively. The hybrid maize varieties used were OBA SUPER-9 (with 105–110 days to maturity) and OBA SUPER-1 (with 105–118 days to maturity) in all the study sites for 2015 and 2016 seasons, respectively. Treatment plot size was 5 m × 6 m (30 m^2^) with a plant spacing of 0.75 m (inter-row) and 0.25 m (intra-row). Detailed information about the nutrient omission trials is provided by Shehu et al. (2018).

### 2.2 Field and laboratory measurement

Four auger soil samples were collected from 0 to 20 cm depths from each experimental field during trial establishment before application of fertilizer treatments using a zig-zag random sampling pattern. The four collected samples were thoroughly mixed to have one disturbed composite sample per experimental field and passed through a 2 mm sieve for laboratory analysis. Total soil organic carbon (OC_tot_) was assessed using a modified Walkley & Black chromic acid wet chemical oxidation and spectrophotometric method (Heanes, 1984). Total nitrogen (N_tot_), was determined using a micro-Kjeldahl digestion method (Bremner, 1996). Soil pH in water (soil/water ratio of 1:1) was measured using a glass electrode pH meter and the particle size distribution with the hydrometer method (Gee and Or, 2002). Available phosphorus (P_av_), available sulphur (S_av_), exchangeable cations (K, Ca, Mg and Na) and micronutrients (Zn, Fe, Cu, Mn and B) were analysed based on the Mehlich-3 extraction procedure (Mehlich, 1984) preceding inductively coupled plasma optical emission spectroscopy (ICP-OES, Optima 800, Winlab 5.5, PerkinElmer Inc.,Waltham, MA, USA). Exchangeable acidity (H + Al) was determined by extracting soil with 1 N KCl and titration of the supernatant with 0.5 M NaOH (Anderson and Ingram, 1993). Effective cation exchange capacity (ECEC) was calculated as the sum of exchangeable cations (K, Ca, Mg and Na) and exchangeable acidity (H + Al).

The crop was harvested at physiological maturity in a net plot of 9 m^2^ (i.e. comprising four middle rows of 3 m length of the experimental plot). Plants in the net plot were harvested, and total fresh weights of cobs and stover were recorded. Ten cobs and five stalks of stover were randomly selected as subsamples for nutrient analysis and to account for grain shelling percentage and moisture content after air-drying. The random selection was carried out by first counting the number of cobs or stalks in the net plot and then randomly arranging them in line; the sub samples were then taken at every interval calculated as the total number of cobs or stalks in the net plot over the number of sub samples to be taken. Finally, grain yield was expressed on a dry weight basis at 15.0% moisture content and the stover yield was expressed on an oven dried basis (dried at 60 °C). The concentration of total nitrogen in the grain and stover was determined using a micro-Kjeldahl digestion method (Bremner, 1996), while P and K were analysed by digestion with nitric acid (HNO_3_) and concentrations measured with inductively coupled plasma optical emission spectroscopy (ICP-OES, Optima 800, Winlab 5.5, PerkinElmer Inc.,Waltham, MA, USA).

### 2.3 Data screening and analysis

The screening of the data was necessitated because some data points were inconsistent and observed to have either soil or plant nutrient concentrations extremely above and below literature range. To address this, multivariate outliers (n = 219) from the experimental data were discarded first at **P < 0.05 using Mahalanobis distance in JMP version 13.0 statistical software (SAS Institute Inc., 2017). Then to understands the characteristics of the screened experimental data (n = 1371), analysis of variance was computed using the same JMP 13.0 statistical software. Nutrient application (NA), agro-ecological zone (AEZ) and variety group (VG) were used as main factors. Season was excluded in the ANOVA because different fields were used between the two seasons of the field experimentation. Mean values with significant differences were compared using Tukey's HSD (Honestly Significant Difference) test. Finally, the screened experimental data was randomly divided into 80% independent fields for parameterization (n = 1090) and the remaining 20% (n = 281) for validation of the QUEFTS model.

### 2.4 QUEFTS model parameterization and validation

#### 2.4.1 Model parameterization

*Step 1 (assessment of the supply of available nutrients)*: the supply of available nutrients (*S*) in the QUEFTS model is given as a function of indigenous soil nutrient supply plus the nutrient input supply. The nutrient input supply is a function of the quantity of nutrient input added multiplied by the average fertilizer recovery efficiency. The indigenous nutrient supply was developed using a multiple regression between soil properties (OC_tot_, N_total_, pH, P_av_ and K) and uptake of the nutrient in the omitted plots using best subset-- selection procedure. The best regression model was chosen based on the highest coefficient of determination value (R^2^) and minimum Bayesian Information Criterion (BIC) among five distribution systems (linear, polynomial, logarithmic, exponential and Cauchy). The fertilizer recovery efficiency (*R_i_*) is then calculated as:

(1) $$R_{i}=(U_{i}-U_{i}^{0})/F_{i}$$

Where *U_i_* = *i*th nutrient in the above ground biomass (kg ha^−1^) in the NPK plot, *U_i_^0^* = *i*th nutrient in the above ground biomass (kg ha^−1^) in the omission plot, *F_i_* = amount of ith nutrient applied (kg ha^−1^).

*Step 2 (relation between the supply of available nutrients and actual uptake)*: The relations between supply of nutrients and actual uptake were calculated using the following conditions and functions (Janssen et al., 1990; Sattari et al., 2014):

(2a) If Si<ri+Sj-rjajdi,then Uij=Si

(2b) If Si>ri+Sj-rj2djai-ajdi, then Uij=ri+Sj-rjdj/ai

(2c) Else  Uij=Si-0.25Si-ri-Sj-rjajdi2Sj-rjdjai-ajdi

Where i,j=N,P, K, i≠j; *U_i_(j)* = refers to uptake of *i*th nutrient in relation to *j*, if *i*= N, j may be P or K; *S_i_* = supply of available *i*th nutrient obtained from step 1; *a_i_* = physiological efficiency (*PhE*) or internal efficiency (*IE*) at maximum accumulation of nutrient *i* (kg grain kg^−1^ nutrient *i*); *d_i_* = physiological efficiency (*PhE*) or internal efficiency (*IE*) at maximum dilution of nutrient *i* (kg grain kg^−1^ nutrient *i*); *r_i_* = minimum nutrient *i* uptake to produce any grain (kg nutrient *i* ha^−1^).

The physiological efficiency (*PhE*) was calculated as follows (Sattari et al., 2014):

(3) $${PhE}_{i}=\frac{1000\times GHI}{GHI\times X_{gi}+\left(1-GHI\right)\times X_{si}}$$

Where G*HI* = grain harvest index, *X_gi_* = mass fraction (g kg^−1^) of the nutrient *i* in the grain, *X_si_* = mass fraction (g kg^−1^) of the nutrient *i* in the stover. The GHI < 0.40 values were considered as anomalies in the dataset as the crop might have suffered biotic and abiotic stresses other than nutrients (Hay, 1995); to guarantee accuracy they were excluded from this analysis.

The minimum uptake of the *i*th nutrient to produce any grain (*r_i_*) was obtained from the minimum uptake of the *i*th nutrient in the above ground biomass mass (kg ha^−1^) in the control plots after discarding all control plots with zero grain yield.

*Step 3 (relation between actual uptake and yield ranges)*: The principles used in QUEFTS at this stage are that the yield ranges are calculated between yield $\left(Y_{i}^{a}\right)$ at maximum accumulation (*a*) and yield $\left(Y_{i}^{d}\right)$ at maximum dilution (*d*), as functions of the actual uptake $\left(U_{i}\right)$ and the minimum uptake to produce any grain $\left(r_{i}\right)$:

(4) $$Y_{i}^{a}=a_{i}\times \left(U_{i}-r_{i}\right),\;i=N,\;P,\;K$$

(5) $$Y_{i}^{d}=d_{i}\times \left(U_{i}-r_{i}\right),\;i=N,\;P,\;K$$

*Step 4 (combining yield ranges to ultimate yield estimates)*: in this final step yield ranges are combined for pairs of nutrients, and then the yields estimated for pairs of nutrients are averaged to obtain an ultimate yield estimate. The following equation was used to calculate yield $\left(Y_{ij}\right)$ for the pair of nutrients *i* and *j* (Sattari et al., 2014):

(6) Yij=Yja+2minYjd, Ykd, Ymax-YjaUi-ri-Yja/diminYjd,  Ykd,  Ymaxai-Yjadi-minYjd, Ykd, Ymax-Yja(Ui-ri-(Yja/di))2minYjd,  Ykd,  Ymaxai-Yjadi2

i,j,k=N,P, K, i≠j≠k; *Ymax* = maximum potential yield (where 10,000 kg ha^−1^ was used in the study area).

The final and ultimate yield estimate $\left(Y_{U}\right)$ is calculated as the mean of the yield estimate of the pairs of nutrients:

(7) $$Y_{U}=\frac{Y_{NP}+Y_{NK}+Y_{PN}+Y_{PK}+Y_{KN}+Y_{KP}}{6}$$

#### 2.4.2 Model validation and sensitivity analysis

The performance of the QUEFTS model was evaluated using four statistical tests i.e. root mean square error (RMSE), coefficient of determination (R^2^), index of agreement and percent bias (PBIAS) (Eqs. (8), (9), (10), (11) below). The RMSE is an error index where the lower the value indicates better model performance (Moriasi et al., 2007). The coefficient of determination (R^2^) estimates the combined dispersion against the single dispersion of the observed and predicted series (Krause and Boyle, 2005); it ranges between 0 and 1, where a value of 0 means no correlation at all and value of 1 means the dispersion of prediction is equal to that of observation. The index of agreement (d) represents the ratio of mean square error and the potential error. The d is interpreted like R^2^ and it has the capability to overcome the low sensitivity of R^2^ to the differences between the observed and predicted means and variances (Legates and McCabe, 1999). The optimal value of PBIAS is 0.00, with low-magnitude values indicating accurate model simulation. Positive values indicate model underestimation bias, and negative values indicate model overestimation bias (Gupta et al., 1999).

The sensitivity analysis was carried out to test the impact of individual parameters and coefficients on model output for each agro-ecological zone and when the data for the two agro-ecological zones were combined to widen the applicability of the model.

(8) $$RMSE=\sqrt{\frac{\sum_{i=1\;}^{n}{\left(Y_{i}^{obs}-Y_{i}^{pre}\right)}^{2}}{n}}$$

(9) $$R^{2}={\left(\frac{\sum_{i=1}^{n}\left(Y_{i}^{obs}-{\bar{Y}}^{obs}\right)\;\left(Y_{i}^{pre}-{\bar{Y}}^{pre}\right)}{\sqrt{\sum_{i=1}^{n}{\left(Y_{i}^{obs}-{\bar{Y}}^{obs}\right)}^{2}}\;\sqrt{\sum_{i=1}^{n}{\left(Y_{i}^{pre}-{\bar{Y}}^{pre}\right)}^{2}}}\right)}^{2}$$

(10) $$d=\frac{\sum_{i=1}^{n}{\left(Y_{i}^{obs}-Y_{i}^{pre}\right)}^{2}}{\sum_{i=1}^{n}{\left({|Y}_{i}^{pre}-{\bar{Y}}^{obs}|+{|Y}_{i}^{obs}-{\bar{Y}}^{obs}|\right)}^{2}}$$

(11) $$PBIAS=\frac{\sum_{i=1}^{n}\left(Y_{i}^{obs}-Y_{i}^{sim}\right)\times \left(100\right)}{\sum_{i=1}^{n}Y_{i}^{obs}}$$

Where $Y_{i}^{obs}$ = *i*^th^ grain yield observed, ${\bar{Y}}^{obs}$ = mean of the observed grain yield, $Y_{i}^{pre}$ = *i*^th^ grain yield predicted by the QUEFTS model, ${\bar{Y}}^{pre}$ =mean of the predicted grain yield and *n* = number of observations.

## 3 Results

### 3.1 Soil characteristics of the experimental fields

There was a strong variability in most soil characteristics among the experimental fields across the two agro-ecological zones (NGS and SS) as indicated by wide range and high coefficient of variability (CV) values (Table 1). However, most of the studied parameters were significantly different between the two agro-ecological zones. Total organic carbon (OC_tot_), total nitrogen (N_tot_), Mg, Cu and available sulphur (S_av)_ were larger in the NGS than in the SS. In contrast, pH, available phosphorus (P_av_), Mn and Fe were larger in the SS than in the NGS. In both agro-ecological zones, soils have a large sand content and are classified as loam in the NGS and sandy loam in the SS. The average soil pH is classified as moderately acidic (5.6–6.0) in the NGS and slightly acidic (6.1–6.5) in the SS. The average contents of OC_tot_ (<10 g kg^−1^), N_tot_ (< 0.10 g kg^−1^), B (< 0.79 mg kg^−1^) and ECEC (< 6.0 cmol_c_ kg^−1^) in both agro-ecological zones fell within a low soil fertility condition according to the ratings of the Nigerian “National Special Programme on Food Security” NSPFS (2005) and of the ESU (1991) fertility classification of Nigerian Savanna soils. However, soil average P_av_ (7–20 mg kg^-1^), K (0.15-0.30 cmol_c_ kg^−1^), Ca (2–5 cmol_c_  kg^−1^), Mg (0.3–1.0 cmol_c_  kg^−1^), Cu (0.21–2.0 mg kg^-1^) and S_av_ (5.1–20.0 mg kg^-1^) were of ‘moderate’ soil fertility status in both agro-ecological zones. High levels of Zn (> 2.0 mg kg^-1^), Mn (> 5.0 mg kg^-1^) and Fe (> 5.0 mg kg^-1^) were observed in the two agro-ecological zones.

**Table 1:** Selected physico-chemical properties of topsoil (0–20 cm) of the experimental fields between the study agro-ecological zones.

Soil Properties	NGS		SS		P-Value
	Mean (Range)	CV (%)	Mean (Range)	CV (%)	
pH_H2O_ (1:1)	5.8 (4.8–7.2)	8	6.2 (5.2–7.2)	9	<0.001**
OC_tot_ (g kg^−1^)	7.25 (2.44–15.45)	36	5.01 (2.04–10.12)	36	<0.001**
N_tot_ (g kg^−1^)	0.47 (0.25–0.98)	30	0.36 (0.17–0.66)	36	<0.001**
P_av_ (mg kg^−1^)	8.43 (0.64–31.77)	82	16.54 (1.44–50.00)	71	<0.001**
Ca (cmol_c_ kg^−1^)	2.31 (0.28–9.78)	43	2.54 (0.38–5.32)	40	0.238
Mg (cmol_c_ kg^−1^)	0.73 (0.07–1.99)	41	0.59 (0.26–1.35)	38	0.011*
K (cmol_c_ kg^−1^)	0.22 (0.06–1.35)	78	0.24 (0.07–0.50)	43	0.499
Na (cmol_c_ kg^−1^)	0.08 (0.04–0.10)	19	0.08(0.04–0.14)	31	0.796
EA (cmol_c_ kg^−1^)	0.04 (0.00–1.00)	7	0.02 (0.00–0.15)	8	0.307
ECEC (cmol_c_ kg^−1^)	3.37 (1.23–11.06)	34	3.45 (1.01–7.17)	36	0.706
Zn (mg kg^−1^)	8.66 (0.83–69.06)	87	9.43 (1.73–37.88)	80	0.589
Cu (mg kg^−1^)	2.00 (0.76–5.12)	47	1.52 (0.76–2.55)	35	0.004**
Mn (mg kg^−1^)	30.9 (3.71–158.46)	65	45.76 (7.49–87.50)	53	<0.001**
Fe (mg kg^−1^)	142.78 (43.36–327.18)	57	207.22(122.87–439.14)	34	<0.001**
B (mg kg^−1^)	0.03 (0.004–0.120)	72	0.02 (0.003–0.100)	112	0.528
S_av_ (mg kg^−1^)	7.29 (4.55–11.70)	20	6.25 (4.09–9.95)	26	<0.001**
Sand (%)	45 (23–70)	20	65 (47–77)	11	<0.001**
Silt (%)	32 (13–59)	22	19 (9–33)	31	<0.001**
Clay (%)	23 (13–42)	24	16 (12–23)	18	<0.001**

NGS: Northern Guinea Savanna; SS: Sudan Savanna; OC_tot_: total organic carbon; N_tot_: total nitrogen; P_av_: available P; S_av_: available sulphur; EA: exchange acidity; ECEC: effective cation exchange capacity; CV: coefficient of variability.

p-value ≤0.01 = highly significant (**) , p-value >0.01 but ≤0.05 = significant (*), p-value >0.05 = not significant.

### 3.2 Characteristics of grain yield and nutrient uptake of the experimental data

**Fig. 3:**
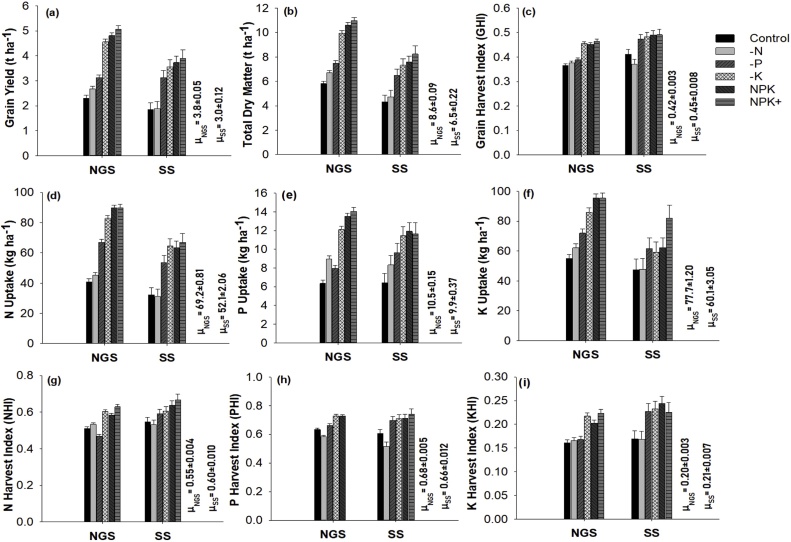
Effects of nutrient application (NA) across agro-ecological zones (AEZ) on (**a**) maize grain yield (**b**) total dry matter (**c**) grain harvest index (**d**) N uptake (**e**) P uptake (**f**) K uptake (**g)** N harvest index (**h**) P harvest index and (**i**) K harvest index. Error bars are standard error of means; NGS: Northern Guinea Savanna; SS: Sudan Savanna; μ_NGS_: mean NGS, μ_SS_ = mean SS.

**Fig. 4:**
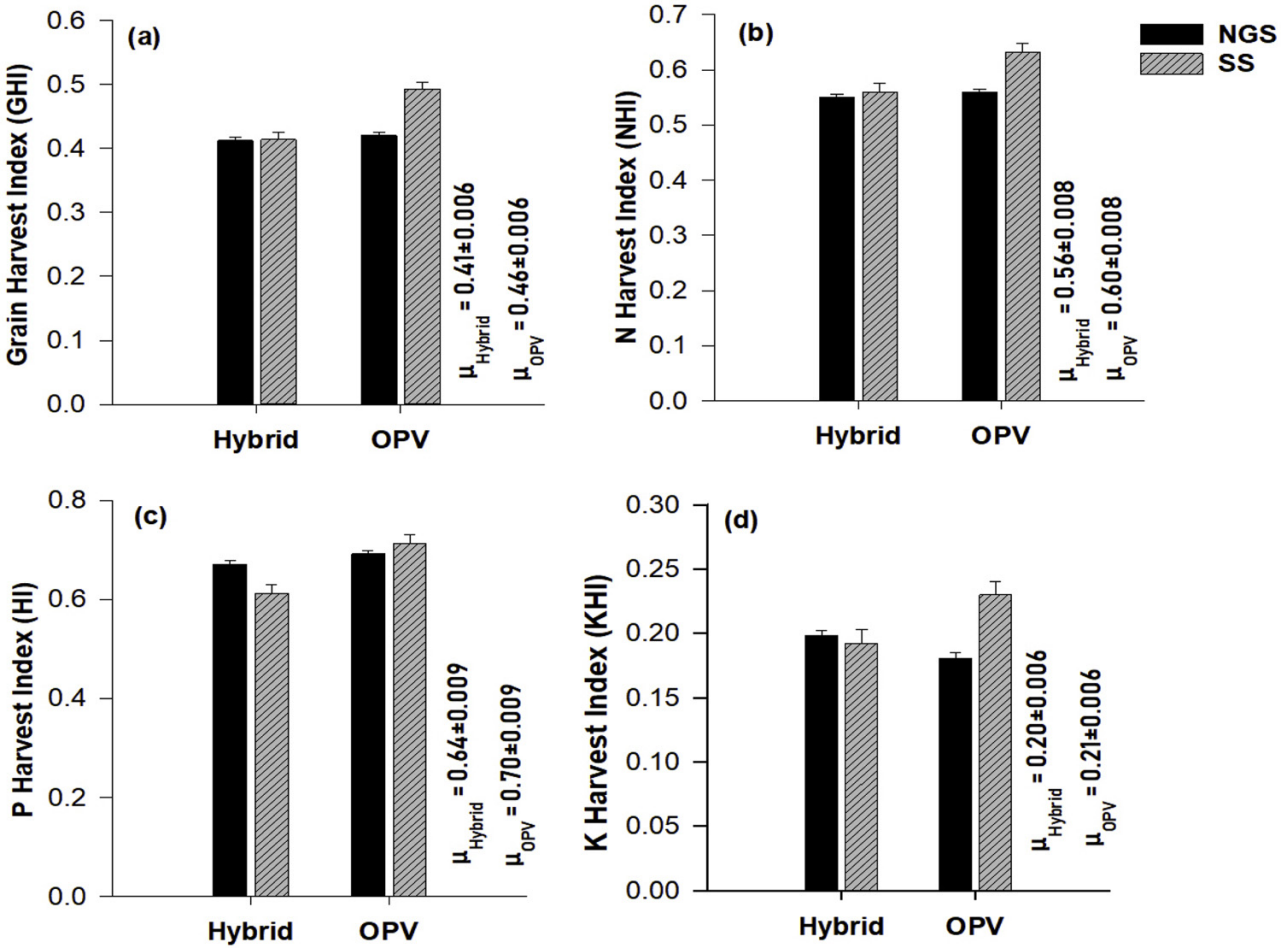
Effects of variety group (VG) across agro-ecological zones (AZE) on (**a**) grain harvest index (**b**) N harvest index (**c**) P harvest index (**d**) K harvest index. Error bars are standard error of means; NGS: Northern Guinea Savanna; SS: Sudan Savanna; OPV: open pollinated variety; hybrid: hybrid variety; μ_Hybrid_: mean hybrid, μ_OPV_ = mean OPV.

Nutrient application (NA) significantly affected all measured grain yield and nutrient uptake characteristics (Table 2). Maize grain yield, total dry matter, N and P uptake were consistently larger in the NPK+, NPK and -K nutrient application treatments than in the -P, -N and control, across the two agro-ecological zones (Fig. 3). Similar trend was observed for grain harvest index (GHI), K uptake and nutrient harvest indices (NHI, PHI, and KHI) except in the SS where the values of these variables for -P treatment were comparable with the values for NPK+, NPK and -K, respectively (Fig. 3). With an exception of plant P uptake (kg ha^−1^) and P harvest index (PHI), all the studied parameters for grain yield and nutrient uptake were significantly different between the agro-ecological zones (AEZ) (Table 2). Grain yield and total dry matter were on average largest in NGS (3.8 and 8.6 t ha^−1^) and smallest in SS (3.0 and 6.5 t ha^−1^) (Fig. 3). Nitrogen (N) and K uptake were equally larger in the NGS (69.2 and 77.7 kg ha^−1^) than in the SS (52.1 and 60.1 kg ha^−1^) (Fig. 3). In contrast, GHI, nitrogen harvest index (NHI) and potassium harvest index (KHI) were larger in the SS than in the NGS. There were few differences between the two variety groups (OPV and hybrid) (Table 2), with only GHI, NHI and PHI being larger in the OPV than in the hybrid variety group (Fig. 4). However, significant interaction among variety group and agro-ecological zone on GHI and N, P and K harvest indices were also observed (Table 2). The GHI was comparable between the two variety groups in the NGS, while in the SS an OPV had larger GHI (0.49) than the hybrid variety (0.41) (Fig. 4). Largest N, P and K harvest indices (NHI, PHI and KHI) were observed in OPV in the SS zone. Because of a few statistical differences between variables of the two variety groups, the datasets from the two groups were used in the parameterization of the QUEFTS model.

**Table 2:** Probability of F values (P-Value) of response of grain yield and nutrient uptake parameters to nutrient application (NA), agro-ecological zones (AEZ) and variety group (VG) of the experimental data.

Parameter	Main Effect			Interaction Effect			
	NA	AEZ	VG	NA x AEZ	NA x VG	AEZ x VG	NA x AEZ x VG
Grain yield (t ha^−1^)	<0.001***	<0.001***	0.634	0.074	0.543	0.571	0.893
Total dry matter (t ha^−1^)	<0.001***	<0.001***	0.103	0.074	0.734	0.176	0.820
Grain Harvest index	<0.001***	<0.001***	<0.001***	0.070	0.174	<0.001***	0.429
Plant N uptake (kg ha^−1^)	<0.001***	<0.001***	0.997	0.173	0.935	0.667	0.882
Plant P uptake (kg ha^−1^)	<0.001***	0.119	0.632	0.077	0.678	0.579	0.894
Plant K uptake (kg ha^−1^)	<0.001***	<0.001***	0.293	0.105	0.679	0.065	0.477
N harvest index	<0.001***	<0.001***	<0.001***	0.016*	0.229	<0.001***	0.168
P harvest index	<0.001***	0.129	<0.001***	0.311	0.125	<0.001***	0.373
K harvest index	<0.001***	<0.001***	0.194	0.183	0.096	<0.001***	0.421

### 3.3 QUEFTS model parameterization

#### 3.3.1 Indigenous soil nutrient supply and fertilizer recovery efficiency

The relations between indigenous soil N, P, and K supply (calculated as the uptake of the given nutrient in the respective omission plots) and soil properties were not effectively described with the QUEFTS’ default equations (Table 3) in each agro-ecological zone and when the data for the two zones were combined as could be derived from the relatively small R^2^ values. Consequently, new and better fitting equations of indigenous soil N, P and K supply were derived for the NGS, SS and the combined zones (Table 3). Total organic carbon (OC_tot_) together with N_tot_ contributed positively as the explaining soil properties for indigenous N soil supply to maize in the NGS. While in the SS and the data of the combined zones only N_tot_ positively explained the indigenous N soil supply. The indigenous soil supply of P in each agro-ecological zone and their combined data were positively explained by pH and P_av_. The exchangeable potassium (K) was the only soil property positively describing the K indigenous soil supply potential to maize in each agroecological zone and across, except in the SS where pH contributed negatively in addition to exchangeable K. The results revealed that unlike in the default QUEFTS model OC_tot_ did not significantly explained the indigenous potential supply of the three macronutrients except N in the NGS.

**Table 3:** Parameterized indigenous maize N, P and K supply equations.

Nutrient	Calibrated	QUEFTS Default (Janssen et al., 1990)
Northern Guinea Savanna (NGS)		
N	S_N_= -20.54 + 0.60 OC_tot_ + 130.92 N_tot_**(R^2^ = 0.57)**	S_N_ = 22.80 + 2.54 OC_tot_**(R^2^ = 0.11)**
P	S_P_ = -12.16 + 2.71 pH + 0.71 P_av_**(R^2^ = 0.61)**	S_P_ = 5.46 - 0.22 OC_tot_ + 0.72 P_av_**(R^2^ = 0.57)**
K	S_K_ = 27.10 + 246.22 K **(R^2^ = 0.55)**	S_K_ = 37.53 - 1.60 OC_tot_ + 248.05 K **(R^2^ = 0.46)**
Sudan Savanna (SS)		
N	S_N_ = 11.64 + 155.41 (N_tot_)^3^**(R^2^ = 0.52)**	S_N_ = 24.87 + 0.61 OC_tot_**(R^2^ = 0.03)**
P	S_P_= -4.11 + 1.40 pH + 0.0005 (P_av_)^3^**(R^2^ = 0.66)**	S_P_ = 3.29 - 0.11 OC_tot_ + 0.31 P_av_**(R^2^ = 0.56)**
K	S_K_ = 228.73 – 35.30 pH + 275.30 K **(R^2^ = 0.60)**	S_K_ = 39.13 - 2.50 OC_tot_ + 237.21 K **(R^2^ = 0.36)**
All (combined agroecological zones)		
N	S_N_ = 9.56 + 147.28 (N_tot_)^2^**(R^2^ = 0.56)**	S_N_ = 22.06 + 2.36 OC_tot_**(R^2^ = 0.10)**
P	S_P_= -8.35 + 2.20 pH + 0.43 P_av_**(R^2^ = 0.50)**	S_P_ = 4.74 + 0.01 OC_tot_ + 0.42 P_av_**(R^2^ = 0.35)**
K	S_K_ = 26.35 + 247.97 K **(R^2^ = 0.52)**	S_K_ = 36.23 – 1.53 OC_tot_ + 248.42 K **(R^2^ = 0.43)**

OC_tot_: total organic carbon (g kg^−1^); N_tot_: total nitrogen (g kg^−1^); Pav: available phosphorus (mg kg^−1^); K: exchangeable potassium (cmol_c_  kg^−1^); pH: soil pH in water (1:1); *S_N_*, *S_P_* and *S_K_* are soil indigenous supplies in kg ha^-1^ of maize crop-available N, P, and K, respectively.

Both the newly parameterized and default QUEFTS average fertilizer recovery efficiencies are shown in Table 4. The fertilizer recovery fractions of N, P and K were substantially larger in the NGS than in the SS (Table 4). In each agro-ecological zone recovery efficiencies of N were smaller than the QUEFTS default value of 0.50. The average P and K recovery efficiencies were larger than the QUEFTS default efficiency values of 0.10 and 0.50, respectively in the NGS and when the data of the two agro-ecological zones were combined. On the contrary, the average P and K recovery efficiencies were smaller than the QUEFTS default values in the SS (Table 4).

**Table 4:** Default and newly parameterized values of average fertilizer recovery efficiency (*R_i_*); physiological efficiency at maximum accumulation of nutrient (*a_i_*) and maximum dilution of nutrient (*d_i_*); and minimum uptake required (*r_i_*) to produce any grain of N, P and K in the above-ground dry matter of maize in the Northern Guinea (NGS), Sudan Savanna (SS) and all (combined data of the two agro-ecological zones).

Coefficients	Nutrients	Default QUEFTS Model (Janssen et al., 1990)	NGS	SS	All
Average fertilizer recovery fraction “*R_i_*”	N	0.50	0.42	0.32	0.40
	P	0.10	0.16	0.08	0.15
	K	0.50	0.54	0.37	0.52

Physiological efficiency at maximum accumulation of the nutrient “*a_i_*” (kg grain kg^−1^ nutrient)	N	30	35	32	35
	P	200	200	164	199
	K	30	25	24	24

Physiological efficiency at maximum dilution of the nutrient “*d_i_*” (kg grain kg^−1^ nutrient)	N	70	79	79	79
	P	600	527	528	528
	K	120	117	136	124

Minimum nutrient uptake to produce any grain “*r_i_*” (kg ha^−1^)	N	5.0	4.0	6.1	4.0
	P	0.4	0.5	0.8	0.5
	K	2.0	4.5	7.3	4.5

#### 3.3.2 Physiological nutrient efficiency and minimum nutrient uptake to produce any grain

**Fig. 5:**
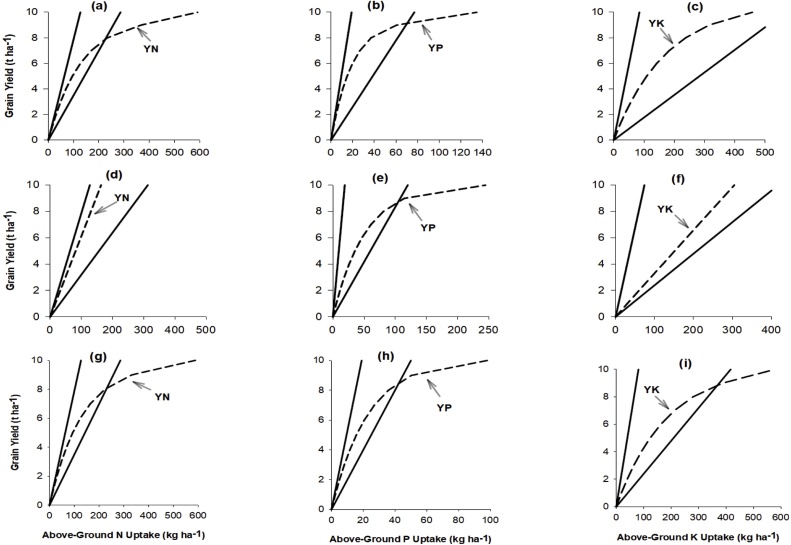
The balanced maize N, P and K uptake requirements (YN, YP and YK) for maximum yield potentials set at 10 t ha^−1^ simulated by the parameterized-QUEFTS model for Northern Guinea Savanna (a–c), Sudan Savanna (d–f) and all i.e. combined data of the two agro-ecological zones (g–i). The upper and lower lines indicate yields with maximum dilution and maximum nutrient accumulation, respectively.

The relations between grain yield and nutrient uptake showing boundary lines of physiological efficiency (*PhE*) of nutrients at maximum accumulation (*a*) and maximum dilution (*d*) are presented in Fig. 5. Across the two agro-ecological zones, the coefficients *a* for N, P and K were overall close to the QUEFTS default values (Table 4). The sole exception was in the SS where coefficient *a* for P was lower than the QUEFTS standard value. The *d* coefficients for N between the NGS, SS and their combined data were comparable but larger than the QUEFTS default value. In contrast, the *d* coefficients for P between the two agro-ecological zones and their combined data were comparable but lower than the QUEFTS default value. The *d* coefficient for K in the NGS and for the data of the combined zones was close to the QUEFTS default value, but these values were lower than the value observed in the SS. The values for the minimum nutrient uptake coefficient (*r*) of N, P and K were 4.0, 0.5 and 4.5 kg ha^−1^ for the NGS and when the data of the two zones were combined; and 6.1, 0.8 and 7.3 kg ha^-1^ for the SS, respectively (Table 4). Across the two agro-ecological zones, the *r* coefficient values for all the three nutrients (N, P, and K) were larger than the QUEFTS default values, except *r* coefficient for the N in the NGS, which was slightly smaller than the QUEFTS default coefficient. However, the *r* coefficient values of the three nutrients were smaller in the NGS than in the SS.

### 3.4 Balanced nutrient uptake requirements

The QUEFTS model predicts a linear relationship between grain yield and above-ground nutrient uptake until yield reaches about 50–60% of the yield potential fixed at 10 t ha^−1^ for the NGS and the SS, respectively (Fig. 5). As the target yield gets closer to the potential yield, *PhE* decreases significantly. The parametrized QUEFTS model estimated a balanced uptake of 21.2 kg N, 3.3 kg P and 23.7 kg K in the above-ground parts per tonne of maize grain yield when the grain yield reached 60% (6 t ha^−1^) of the maize potential yield in the NGS (Table 5). The corresponding *PhE* was 52.6 kg grain kg^−1^ N, 337.5 kg grain kg^−1^ P and 45.8 kg grain kg^−1^ K. In the SS an uptake of 16.3 kg N, 7.7 kg P and 30.4 kg K was required per tonne of grain yield at 60% of the potential yield (Table 5); the corresponding *PhE* was 61.5 kg grain kg^−1^ N, 142.4 kg grain kg^−1^ P and 33.0 kg grain kg^−1^ K. Likewise, when the data of the two agro-ecological zones were combined an uptake of 20.7 kg N, 3.4 kg P and 27.1 kg K are required to produce 1 t maize grain when at 60% of the potential yield; this corresponds to *PhE* of 48.4 kg grain kg^−1^ N, 290.8 kg grain kg^−1^ P and 36.9 kg grain kg^−1^ K. It follows that the optimal N, P & K ratios in the above-ground dry matter at 60% of the maize potential yield are 6.4:1:7.2 for the NGS, 2.1:1:3.9 for the SS and 6.1:1:7.9 when the data of two zones were combined. These results show that the QUEFTS model predicts larger P and K uptake requirements for a balanced nutrition at 60% of the potential yield in the SS than in the NGS, while an opposite trend was observed for N requirements between the two agro-ecological zones.

**Table 5:** Maize reciprocal physiological efficiency (*RPhE*) of N, P, and K simulated by the QUFETS model to achieve yield targets with maximum yield potential set at 10 t ha^−1^ for the Northern Guinea Savanna (NGS), Sudan Savanna (SS) and all (combined data of the two agro-ecological zones).

Yield (t ha^−1^)	NGS RPhE (kg nutrient t^−1^ grain)			SS RPhE (kg nutrient t^−1^ grain)			All RPhE (kg nutrient t^−1^ grain)		
	N	P	K	N	P	K	N	P	K
0	0.0	0.0	0.0	0.0	0.0	0.0	0.0	0.0	0.0
1	14.2	2.2	17.1	16.2	5.4	30.2	14.0	2.4	19.0
2	15.1	2.3	18.0	16.3	5.7	30.2	14.9	2.5	20.1
3	16.1	2.5	19.0	16.3	6.0	30.3	15.9	2.7	21.3
4	17.4	2.7	20.3	16.3	6.5	30.3	17.1	2.9	22.9
5	19.0	3.0	21.8	16.3	7.0	30.3	18.7	3.1	24.7
6	21.2	3.3	23.7	16.3	7.7	30.4	20.7	3.4	27.1
7	24.2	3.8	26.2	16.3	8.7	30.4	23.5	3.9	30.3
8	29.2	4.6	29.6	16.3	10.1	30.4	27.8	4.5	35.0
9	40.8	6.7	35.0	16.3	12.7	30.4	36.9	5.6	43.1
10	59.1	13.5	45.8	16.3	24.5	30.5	58.8	9.8	57.8

### 3.5 QUEFTS model validation and sensitivity analysis

**Fig. 6:**
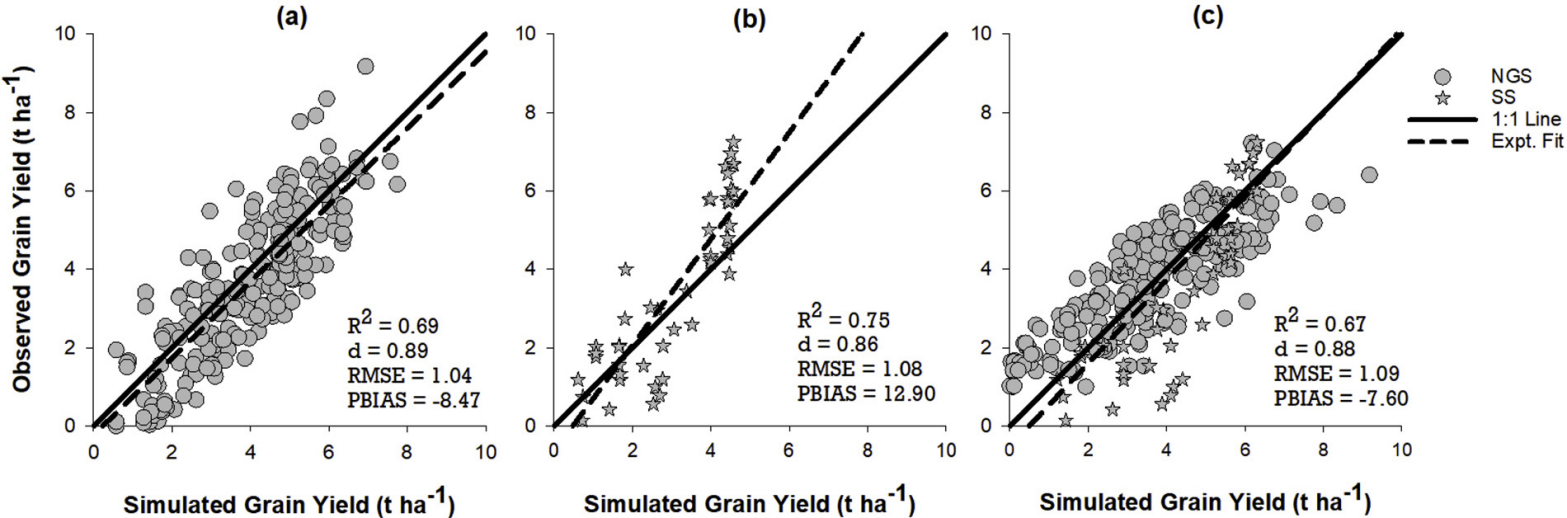
Relation between the observed and parameterized QUEFTS simulated maize grain yield for (a) Northern Guinea Savanna “NGS”, (b) Sudan Savanna “SS” and (c) for the all (combined data of the two agro-ecological zones). R^2^: coefficient of determination; d = index of agreement; RMSE: root mean square error (t ha^−1^); PBIAS: percent bias (%).

Fig. 6 shows the comparison between observed and parameterized QUEFTS predicted maize grain yields for the NGS, SS and for the combined data of the two agro-ecological zones. There was a satisfactory agreement between grain yields predicted by the parameterized QUEFTS model and those observed from the field experiment in each agro-ecological zone (owing to reasonably high R^2^ and d values and relatively small RMSE) (Fig. 6a and b). However, the model showed a small overestimation bias in the NGS (PBIAS = -8.5%) and a small underestimation bias in the SS (PBIAS = 12.9%).

The sensitivity analysis shows the performance of the model to be slightly reduced when the data of two agro-eclogical zones were combined (indicated by 2% and 8% reduction in R^2^ value over NGS and SS alone, respectively). However, the parameterized model for the data of the combined agro-ecological zones similarly displayed small overestimation bias of 7.6% (Fig. 6c).

## 4 Discussion

### 4.1 Soil characteristics of the experimental fields

The larger OC_tot_, N_tot_ and S_av_ in NGS is associated with larger soil clay content as well as longer rainy season which resulted in greater vegetative biomass and litterfall than in SS. In contrast, relatively less rainfall in the SS resulted in higher average pH values compared to the NGS. High rainfall increases the potential for leaching of cations (especially Ca and Mg) and poor soil aeration, which often decreases soil pH. The higher OC_tot_, N_tot_ and S_av_, and lower pH in the NGS than in the SS has also been observed by Daudu et al. (2018). Northern Nigerian Savanna soils are developed from aeolian materials and pre-Cambrian basement complex rocks (such as granite, schist and sandstone) (Bennett, 1980) and this resulted in a large sand fraction in the surface soils of both the NGS and the SS. Moreover, Malgwi et al. (2000) and Voncir et al. (2008) have reported that sorting of soil material due to clay eluviation and wind erosion as additional factors leading to large sand content of the surface soils of the Northern Nigerian Savanna. The overall low levels of OC_tot_, N_tot_, B and ECEC in both agro-ecological zones have been related to two principal factors: (i) the type of parent material and intensive weathering of the soils with small mineral reserve necessary for inherent nutrient recharge; and (ii) intensive cultivation of the soils with inadequate (unbalanced and insufficient external inputs) nutrient management including burning or complete removal of crop residues (Jones and Wild, 1975; Manu et al., 1991; Smaling et al., 1991; Kwari et al., 2011). Although past studies like Ekeleme et al. (2014); Kamara et al. (2014) and Shehu et al. (2015) have reported low average P_av_ in some parts of the study area, the moderate average P_av_ content observed in the two agro-ecological zones in this study can be explained by residual effects of previous P applications. Some fractions of P applied through fertilizer not taken up by the current crop due to temporary fixation in the soil can be released gradually to the succeeding crop (Janssen et al., 1987). The moderate to high levels of exchangeable cations (Ca, Mg and K) are not surprising as most soils are developed from basement complex rocks which contains large content of these cations. Møberg and Esu (1991) also reported an appreciable presence of K-bearing feldspar minerals in sand and silt particles of Savanna soils of Nigeria. Similarly, deficiency of the studied micronutrients (Fe, Mn, Cu and Zn) is unlikely to occur in the study fields due to relatively acidic reaction of the soils. Only at pH above 7.5 does the availability of these micronutrients becomes significantly limited owing to the formation of oxides, hydroxides and carbonates (Sillanpää, 1982).

### 4.2 Characteristics of grain yield and nutrient uptake of the experimental data

The minimal response of grain yield, total dry matter, GHI and N, P and K uptake in control, -N and -P relative to the NPK+, NPK and -K treatments across the two agro-ecological zones indicates N and P as the major nutrients limiting growth and yield response of maize. Nitrogen deficiency has been recognized as the most limiting factor for cereal production in vast areas of SSA including in the Nigerian Savanna (Vanlauwe et al., 2011). Soil N can be depleted rapidly by maize, especially when yields are high and stover is exported (Kamara, 2017). The widespread N deficiency in the study area can be attributed to small soil organic matter contents (indicated by small OC_tot_) resulting from inherent poor soil fertility and continuous cropping with inadequate and imbalanced N fertilizer or manure applications. Adediran and Banjoko (1995) reported P as among the most maize yield limiting nutrients in the Nigerian Savanna. Nigerian soils, particularly the highly weathered ones, have small indigenous P contents and often a large P sorption capacity (Osemwotai et al., 2005). Combined application of balanced fertilizers with manure and rotation of cereal crops with legumes through integrated soil fertility management principles (ISFM) (Vanlauwe et al., 2010) can assist farmers in the study area to improve soil N and P status. The lack of a significant increase in grain yield due the addition of secondary macronutrients (S, Ca and Mg) and micronutrients (Zn and B) suggest that these nutrients are not significantly limiting maize yield in the studied area. A significant extra yield increases due to the addition of the secondary macronutrients and micronutrients (SMMs) was observed in only 7 fields (Shehu et al., 2018). The lack of large yield response to the addition of the SMMs did not support the findings of Wendt and Rijpma (1997) who reported large improvement in maize yield in some parts of East Africa due to the addition of the SMMs and recommended inclusion of the SMMs in NPK fertilizer blends. The larger grain yield and total dry matter in the NGS compared with the SS could be explained by the amount of rainfall, as the larger relative rainfall amount and duration in the NGS favoured more maize biomass production than in the SS.

#### 4.2.1 Indigenous soil nutrient supply and fertilizer recovery efficiency

The newly developed supply functions for indigenous soil N, P and K in both agroecological zones explained a minimum of 50% variation in soil characteristics among the studied fields. The unexplained variation can be attributed to the differences in rate of mineralization, in leaching losses and in soil moisture availability, etc. (Barber, 1995). These remain complex factors to integrate into a simple empirical indigenous nutrient supply equation (Tabi et al., 2008). Going beyond the default QUEFTS model, total nitrogen (N_tot_) represents a more apt explanatory variable for the indigenous soil supply of nitrogen (S_N_) rather than the conventional OC_tot_. Nitrogen mineralization in soil is indeed directly related to microbial activity and organic matter inputs, which are influenced by a combination of several physical, biological and chemical factors in the soil system (He, 2014). Hence, it is no surprise that OC_tot_ does not consistently provide the best proxy for N-availability in the soil. Comparable to this study, Samaké (2003) also reported OC_tot_ did not statistically influence indigenous supply of N, P and K to pearl millet in the similar soil conditions in Mali. The effect of pH on indigenous soil supply of P (S_P_) across the agro-ecologies corroborates findings of Janssen et al. (1990). Most of the studied fields have acidic pH values, at this condition a unit decrease in pH level increases the potential of conversion of available phosphorus into a less soluble form through reacting with Al and Fe.

Favourable combinations of adequate rainfall and low night temperatures makes the NGS more suitable for maize production than the SS (Badu-Apraku et al., 2015), this translates into the larger N, P and K fertilizer recovery efficiencies observed in the NGS. Despite in overall N and P recovery efficiency (R_N_ and R_P_) fell below the default QUEFTS values across the two agro-ecologies, but the values in the NGS are close to the result obtained by Saïdou et al. (2003) of 0.40 and 0.14 for N and P, respectively in the Southern Benin. In the same way, the recovery efficiency of K (R_K_) in the SS is in agreement with 0.40 reported in the Southern Benin by the same Saïdou et al. (2003). However, the R_P_ of both NGS and SS is smaller than the value of 0.24 observed by Tabi et al. (2008) in some part of the Northern Nigeria. This suggest that effective results which optimize fertilizer recovery efficiency figures can be obtained exclusively if site-specific nutrient recommendations using balanced nutrient requirements are complemented with the right source, time and placement of fertilizer application, and subject to appropriate agronomic practices.

#### 4.2.2 Boundary line coefficients for physiological efficiency of nutrients and minimum nutrient uptake to produce any grain

The boundary line coefficients *a* and *d* for physiological nutrient efficiency of this study across the two agro-ecological zones are larger than in the analysis of Saïdou et al. (2003) in the Southern Benin (20 and 40 kg grain kg^−1^ N, 110 and 270 kg grain kg^−1^ P, 25 and 90 kg grain kg^−1^ K) except *a* coefficients for K that are comparable. Equally, Tabi et al. (2008) observed smaller *a* and *d* boundary line physiological efficiency for N and P in some part of Northern Nigeria (21 and 71 kg grain kg^−1^ N, 97 and 600 kg grain kg^−1^ P) except *d* coefficient for P that is larger compared with the values of this study. Saïdou et al. (2003) and Tabi et al. (2008) have attributed the smaller physiological efficiencies in their studies to smaller grain harvest indices. Therefore, the larger values of physiological efficiencies in this study proved to be the result of large grain harvest indices. As explained earlier under sub-section 2.4.1, grain harvest indices less than 0.40 were considered as anomalies in the dataset as the crop might have suffered biotic and abiotic stresses other than nutrients (Hay, 1995); to guarantee precision were excluded as similarly performed by Liu et al. (2006), Xu et al. (2013), among others.

The significant difference between the minimum uptake requirement to produce any grain *(r*) observed in this study and the QUEFTS default values emphasizes the importance for recalibration of this parameter which has not been considered in most previous QUEFTS parameterization and calibration studies.

### 4.3 Balanced nutrient uptake requirements

Balanced nutrient plant uptake requirement can provide guidance for amount of fertilizer to be applied to achieve a desirable yield and for an efficient maintenance of soil fertility, as at least the nutrients removed or harvested in the above ground plant dry matter must be returned to the soil. The balanced nutrient uptake requirements predicted by QUEFTS in this study with exception of K in the SS are comparable to values of 20.0 kg N, 4.5 kg P, 18.0 kg K reported for a tonne of maize grain in similar environmental and soil conditions in Zimbabwe (Piha, 1993). However, the higher balanced K uptake ratio in the above-ground matter relative to N as predicted by the parameterized QUEFTS in this study across the two agro-ecologies does not support the findings of most previous studies which have reported higher N uptake ratio compared to K. This trend was not surprising as most of the study fields have moderate to high K content in addition to the amount K fertilizer applied of 40-50 kg K ha^−1^. This led to luxury uptake of K especially in the maize stover evidenced by a small K harvest index (KHI). The moderate to high K content of the soils could be linked to an appreciable amount of K-bearing feldspar minerals in the sand and silt particles in the study area (Møberg and Esu, 1991) and the residual effect of previous K fertilizer applications. The supply of available K in soil is strongly dependent upon the type and amount of K-bearing minerals. In the K-feldspars, K is structurally bound in the crystal lattice (structural K) and is only released into the soil solution through weathering (Øgaard and Krogstad, 2005). The larger P uptake requirements in SS relative to the NGS can be attributed to higher soil P content in the SS as confirmed by the low maize yield response to P application observed in the nutrient omission trials.

### 4.4 QUEFTS model validation and sensitivity analysis

The close agreement between the parametrized QUEFTS simulated and observed yields shows that the parameterized QUEFTS model can be used to calculate balanced nutrient requirements and site- or area-specific fertilizer recommendations to optimize maize yield in the Northern Nigerian Savanna. The QUEFTS model, however, assumes that other biophysical factors apart from nutrients such as moisture, temperature, pests, diseases and management are non-limiting. As these factors are complex to optimize in on-farm field experiments, this may account for the under- and over-estimation bias obtained with the parameterized QUEFTS model in the SS and the NGS, respectively. To guarantee precision, the under- and over- estimation percent bias in the SS and NGS, respectively should be considered and adjusted at the final and ultimate yield estimate $\left(Y_{U}\right)$ stage in the parameterized QUEFTS model. The good performance of the model when data for the two agro-ecological zones were combined suggests that the parametrized nutrient supply functions and other calibrated parameters can be widely adopted for a larger scale application.

## 5 Conclusion

The present study resulted in the parameterization and validation of the QUEFTS model to arrive at balanced nutrient requirements and site-specific fertilizer recommendations for maize in the Northern Nigerian Savanna. This was based on data from on-farm nutrient omission trials conducted across potential maize production sites covering two agro-ecological zones i.e. the Northern Guinea Savanna (NGS) and the Sudan Savanna (SS). There were considerable differences in soil and nutrient uptake characteristics between the NGS and the SS. The relations between indigenous soil N, P, and K supply and soil properties were not adequately described with the QUEFTS default equations across the agro-ecological zones, consequently new and better fitting equations were derived. The coefficients *a* and *d* of N, P, and K for the QUEFTS model were 35 and 79, 200 and 527, and 25 and 117 kg grain kg^−1^ nutrient for the NGS; 32 and 79, 164 and 528, and 24 and 136 kg grain kg^−1^ nutrient for the SS zone; and 35 and 79, 199 and 528, and 24 and 124 kg grain kg^−1^ nutrient when the data of the two agro-ecological zones were combined. The minimum nutrient uptake coefficients (*r*) of N, P and K were 4.0, 0.5 and 4.5 kg ha^-1^ for the NGS zone and the combined data of the two agro-ecological zones; and 6.1, 0.8 and 7.3 kg ha^−1^ for the SS zone. The parameterized QUEFTS model predicted a linear increase in above-ground dry matter uptake of N, P and K until the grain yield reached about 50–60% of the potential yield. At 60% of the potential yield (6 t ha^−1^) a balanced uptake in the above-ground part of 21.2 kg N, 3.3 kg P and 23.7 kg K is required to produce a tonne of maize grain in the NGS; 16.3 kg N, 7.7 kg P and 30.4 kg K to produce a tonne of maize grain in the SS zone; and 20.7 kg N, 3.4 kg P and 27.1 kg K to produce a tonne of maize grain when the data of the two agro-ecological zones were combined. Validation results indicated a good correlation between the parameterized QUEFTS estimated and observed grain yields in both agro-ecological zones. The sensitivity analysis revealed that the calibration parameters obtained across the two agro-ecological zones did not substantially reduce the precision of the model when compared with those obtained from the data of the individual agro-ecological zone. This imply that the parametrized QUEFTS model can be a springboard for development of simple and cost-effective decision support tools for nutrient management and fertilizer recommendations in the Northern Nigerian Savanna and in similar environments of West and Central Africa. To ensure a greater impact, site-specific fertilizer recommendations developed from the model must be complemented with appropriate agronomic management practices including use of right source, precise time and right placement of the fertilizer.
